# The AUTNES Online Panel Study 2017–2024: A Dataset of Austrian Voter Attitudes and Behavior

**DOI:** 10.1038/s41597-025-05848-2

**Published:** 2025-08-26

**Authors:** Julia Partheymüller, Sylvia Kritzinger, Markus Wagner

**Affiliations:** University of Vienna, Department of Government, Kolingasse 14–16, 1090 Vienna, Austria

**Keywords:** Politics, Government

## Abstract

The AUTNES Online Panel Study, a component of the Austrian National Election Study (AUTNES), is a longitudinal survey designed to analyze electoral dynamics in Austria across multiple election cycles, spanning from 2017 to 2024. This multi-wave online panel combines repeated measures of core variables to enable over-time analysis with thematic modules and survey experiments for an in-depth examination of political behavior, attitudes, and voter psychology. This paper offers a comprehensive overview of the study’s research design and methodology, evaluating the quality of the collected data with a focus on representativeness and panel participation. The assessment aims to equip data users with a detailed understanding of the dataset and underscore its potential for longitudinal analyses.

## Background & Summary

Elections are a cornerstone of democratic systems, as they embody the mechanism through which citizens express their political preferences and influence governance, while at the same time being critical moments for politicians and political parties^1,2^. According to Sartori, elections constitute the only moment when the people in a representative democracy indeed rule^3^. They serve as key junctures for understanding political stability and change. Recognizing their fundamental role, scientific research has long prioritized the systematic study of voter attitudes, beliefs, and behaviors.

Structured election research originated in the United States with large-scale projects such as the American National Election Studies (ANES) and has since gradually expanded to countries worldwide. In Austria, systematic and continuous electoral research began with the Austrian National Election Study (AUTNES), established in 2009 following the 2008 parliamentary election. Elections constitute a critical moment for gathering data: if these moments pass without collecting empirical data, then information on voter perceptions, attitudes and behavior cannot be generated at a later point in time and is thus lost forever. Data on Austrian voters in parliamentary elections are thus not available prior to 2009 and the existence of AUTNES. This is not to say that no survey data was collected for previous elections, but these were collected by political parties for their own policy and campaign purposes, were limited in scope and purpose, and – most importantly – not available for academic research.

In this context, AUTNES was established and institutionalized as a comprehensive research program that aimed to enhance the understanding of Austrian democracy by examining the dynamics of elections, voter behavior, and political processes. AUTNES innovated by integrating multiple perspectives on elections: the demand side (voters), the supply side (parties and candidates), and the mass media as the link between the two. The leading aim of AUTNES was to connect research on key electoral actors and enable the integration of various data sources, including survey data and text data from party communication and media coverage. As a result, AUTNES went beyond the collection of survey data and also gathered text data from party communication and media outlets. Through the collection of high-quality, systematic data on voters, parties, and the media, AUTNES contributes to academic, public, and political discourse on democratic practices in Austria. Moreover, in integrating these data sources AUTNES also provides a blueprint for other national election studies on how to develop and link data on the demand side with data from the media and supply side. In a nutshell, AUTNES allowed researchers with dispersed expertise in the study of voters, parties, and the media to coordinate efforts, with the aim of creating a whole that is much more than the sum of its parts.

This data paper focuses on the voter component, specifically the AUTNES Online Panel Study (2017–2024)^4^, a key part of the AUTNES infrastructure. Similar to the British Election Study Internet Panel (2014–2024)^5^ and the GLES Panel (2016–2021)^6^, this voter panel survey has been tracking public opinion over an extended period. The paper outlines the study’s research design and methods, assesses data quality, and thereby aims to enhance accessibility for a broad range of users. By tracking the evolution of political attitudes and behaviors at the individual level, the panel study documents public sentiment during pivotal moments in recent Austrian political history, serving as a valuable resource for scholars, students, policymakers, and engaged citizens.

## Methods

The AUTNES Online Panel Study is a comprehensive longitudinal multi-wave online panel survey designed to analyze public perceptions, political attitudes, and voting behavior among Austrian citizens^4^. By re-interviewing the same respondents over time, panel designs track individual-level change, capture shifts in attitudes, and identify causal relationships, providing more accurate data and deeper insights into social and political dynamics than cross-sectional surveys^7^. The study focuses on individuals aged 16 and above who are eligible to vote, with 16 being the legal voting age in all elections in Austria since 2007^8^. Since its launch in 2017, the panel survey has conducted 23 waves, covering all nationwide elections during this period, including the Austrian parliamentary elections of 2017, 2019, and 2024, the European Parliament elections in 2019 and 2024, and the Austrian presidential election in 2022. Each wave involved approximately 3,000 respondents, and the surveys were administered online (Computer-assisted web interviews, CAWI).

Survey respondents were recruited from Austria’s largest commercial non-probability online access panel, maintained by Marketagent GmbH, which has 154,000 enrolled members and is certified under ISO 20252^9^. The survey institute employs a cross-media enrollment strategy, integrating offline channels (TV, radio, print, and billboards) with online methods (social media campaigns, influencer marketing, search engine advertising, and affiliate partnerships) to ensure broad and representative participant recruitment. Marketagent ensures high participant quality through certified panel management and rigorous quality assurance standards.

From this online access pool survey respondents were recruited using representative quotas based on demographic targets from the Austrian Mikrozensus (Statistics Austria), a large-scale household survey providing key demographic and socio-economic information about the Austrian population. The quota criteria included age, gender, age-gender interaction, education, household size, region, and population size to achieve a high level of representativeness of the survey participants for the target population of Austrian eligible voters. All research participants gave their informed consent when they registered to join the survey pool. The participation in the study was entirely voluntary, and participants were free to withdraw from the study at any point.

**Fig. 1:**
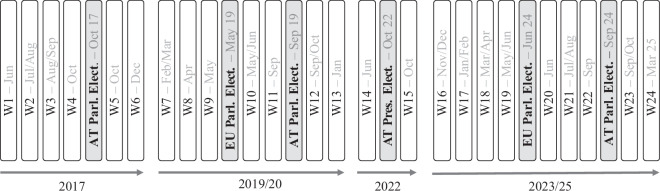
Schematic Overview of the Schedule of Waves.

The AUTNES Online Panel Study 2017–2024 waves were scheduled around key elections to capture shifts in attitudes and behaviors (see Fig. 1)^4^. The first six waves focused on the 2017 parliamentary election, with four conducted before and two after the election, including one following government formation. The election led to a rightward shift in Austrian politics, resulting in a coalition between the center-right Austrian New People’s Party (ÖVP), led by Chancellor Sebastian Kurz, and the populist radical-right Austrian Freedom Party (FPÖ)^10^. In 2019, the survey initially focused on the European Parliament election but was expanded to include the parliamentary election after the government had unexpectedly collapsed during fieldwork due to the so-called Ibiza scandal^11^. The parliamentary election that followed resulted in the formation of an ÖVP-Greens coalition in January 2020. After a pause during the first years of the COVID-19 pandemic, two waves were conducted in the context of the 2022 presidential election, capturing the reelection of President Alexander van der Bellen. Fieldwork was then resumed again in November 2023 and continued through the 2024 European Parliament and the Austrian parliamentary elections later that year, from which the FPÖ emerged as the leading party, securing the most votes. A final wave was conducted after the record-long coalition negotiations were completed in March 2025^12^ and is in the process of being added to the dataset.

The fieldwork for each wave typically lasted two to three weeks, with regular reminders being sent out after the initial invitation to encourage participation. While the primary goal was typically to contact all respondents as quickly as possible, the pre-electoral survey waves leading up to the Austrian and European Parliament elections in 2019 and 2024 (Waves 9, 11, 19, and 22) were systematically distributed over the field period. This approach followed the principles of a rolling cross-section design to capture potential campaign effects and the influence of unexpected events occurring during fieldwork^13,14^. Hence, the data for these four elections not only allow for comparisons between panel waves but also enable the analysis of within-campaign dynamics, providing a more granular understanding of short-term shifts in public opinion.

The questionnaire was designed to track key aspects of political behavior and public opinion over time. It included questions on voting behavior, such as vote choice in past elections and current vote intentions. In addition, the survey measured longstanding political predispositions such as party identification, left-right ideology, and trust in democratic institutions, as well as populist attitudes. It also captured short-term factors including political attitudes toward parties, candidates, policy issues, and government performance, along with media consumption, covering both traditional and new media sources. Core questions were repeated across waves to enable longitudinal comparisons, while new question modules were introduced to explore context-specific themes during each electoral cycle, such as perceptions of electoral integrity, attitudes toward EU integration, migration and social groups, as well as toward taxation and social spending.

The thematic modules often also included survey experiments designed to test specific hypotheses and theoretical mechanisms. Survey experiments often involved simple split-ballot designs but sometimes also conjoint designs, exploring broader themes such as the role of emotions in politics, attitudes toward welfare reforms, political scandals and blunders, the influence of polls, and various factors shaping perceptions and support for politicians and parties. Prior fieldwork survey experiments underwent ethical screening and were approved by the Institutional Review Board of the Faculty of Social Sciences at the University of Vienna. Socio-demographic information, including age, gender, education, region, and household size, was collected to facilitate subgroup analyses. Socio-demographic information was usually captured in the entry wave when participants initially joined the study and updated only across longer intervals, as socio-demographic characteristics can be expected to be mostly stable in the short-run.

Completing the questionnaire took between 15 and 25 minutes, depending on its complexity. Respondents received a financial incentive of 200 credit points (equivalent to 2 Euro) per completed interview to maintain high participation rates across the study period.

## Data Records

The AUTNES Online Panel Study 2017–2024 is available from the AUTNES Dataverse at the Austrian Social Science Data Archive (AUSSDA, www.aussda.at, doi:10.11587/HNUFCC)^4^. AUSSDA is a certified national research infrastructure providing sustainable and user-friendly digital archiving services for researchers, students, and media professionals. AUSSDA ensures research data are findable, accessible, interoperable, and reusable (i.e., the FAIR principles)^15^, while promoting open science and upholding data protection and ethical standards. As Austria’s representative in the Consortium of European Social Science Data Archives (CESSDA), a European Research Infrastructure Consortium (ERIC), AUSSDA operates across multiple university locations and collaborates with national and international partners.

The repository of the AUTNES Online Panel Study 2017–2024 contains the full panel dataset in various formats, available in both the German original and English translations, along with the full questionnaires and comprehensive documentation to support scientific research^4^.

Data Files (6221 variables, 11,184 observations):

- STATA: German (10874_da_de_v1_0.dta), English (10874_da_en_v1_0.dta)
- R: German (10874_da_de_v1_0.Rdata), English (10874_da_en_v1_0.Rdata)
- CSV: German (10874_da_de_v1_0.csv), English (10874_da_en_v1_0.csv)
- SPSS German (10874_da_de_v1_0.zsav), English (10874_da_en_v1_0.zsav)

Questionnaires:

- German: 10874_qu_de_v1_0.pdf
- English: 10874_qu_en_v1_0.pdf

Documentation:

- Method Report (English): 10874_mr_en_v1_0.pdf
- Variable List (English and German): 10874_om_en_v1_0.xlsx

The files of the AUTNES Online Panel Study 2017–2024 are available under specific licensing conditions^4^. The data files can be used exclusively for scientific purposes and users are required to confirm to comply upon download. The documentation files, including questionnaires, variable lists, and the method report, are provided under a Creative Commons Attribution 4.0 International License (CC BY 4.0), allowing easy access and broader reuse with proper attribution.

## Technical Validation

### Accuracy

In survey analysis, a common concern is the accuracy with which the survey sample aligns with the socio-demographic and political characteristics of the target population, in our case the citizens eligible to vote in Austrian parliamentary elections. Ensuring accuracy is essential as it supports the credibility of inferences about population trends, political attitudes, and electoral behavior drawn from the data. Deviations from target distributions can arise, particularly for populations that are traditionally more difficult to reach in online or political surveys, such as individuals of old age, those with low education, migration backgrounds, or non-voters.

**Fig. 2:**
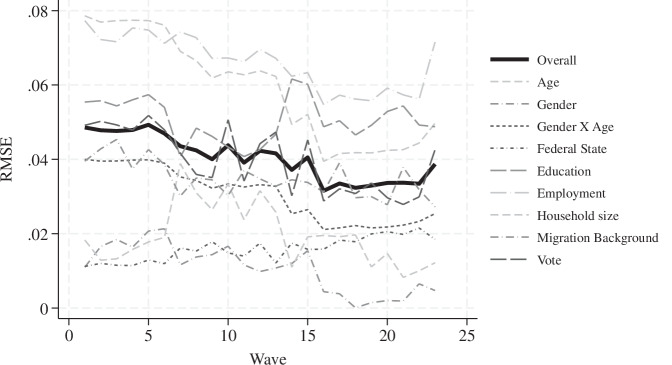
Accuracy of the Raw Unweighted Data Versus Population Targets.

Accuracy is commonly assessed using the Root Mean Squared Error (RMSE), which quantifies deviations between observed values and target distributions^16^. As illustrated in Fig. 2, the raw data from the AUTNES Online Panel Study^4^ demonstrate high overall accuracy, with the average RMSE across all categories typically below 0.05, corresponding to an average deviation of 5 percentage points from the target.

Initial deviations were most pronounced for age and employment status due to underrepresentation of the oldest age cohorts and pensioners, but still these deviations remained below 0.08 averaged across the age groups. Over successive survey waves, the RMSE decreased, reflecting improved sample accuracy, primarily through better coverage of older age cohorts and retirees, indicating that the online panel became increasingly better in capturing all age groups. Due to this trend, the raw sample composition in more recent waves aligns increasingly closely with the target distributions. A comprehensive methodological comparison of the 2024 post-election wave of the AUTNES Online Panel Study and the probability-based Digitize! CSES Post-Election Survey^17^ further shows that the AUTNES non-probability panel achieved comparable or higher accuracy on key sociodemographic and political indicators and was also more effective in reaching non-voters and individuals with very low levels of political interest^18^.

To further enhance accuracy and representativeness, weights were calculated using Iterative Proportional Fitting (IPF)^19^. Weights adjust the sample to match the following target distributions:

- Age: 6 levels
- Gender: 2 levels
- Age X Gender: 12 levels
- Education: 5 levels
- Federal State: 9 levels
- Employment Status: 4 levels
- Household Size: 3 levels
- Migration Background: 2 levels
- Vote Recall (incl. turnout and party choice): 7-8 levels

The dataset includes two weighting variables for post-stratification adjustment to known population distributions:

- Demographic weight (w*_weightd): The “demographic” weight adjusts the sample to known socio-demographic population distributions. These weights are ideal for consistent comparisons across time.
- Demographic + political weight (w*_weightp): The “political” weight further adjusts for turnout and vote choice marginals using reported voting behavior in the most recent elections. These weights are particularly suited for post-election analyses to accurately reproduce official election results.

Weighting variables were computed using the Stata module “ipfweight”, with values constrained to a minimum of 0.2 and a maximum of 5.0^20^. Missing values in the target variables were weighted neutrally. The target values are based on the Austrian *Mikrozensus* and official election results provided by the Austrian *Federal Ministry of the Interior* (BMI). Across the 23 survey waves, the design effect of the demographic weights ranges from 1.26 to 1.81, with an average of 1.46. For the combined demographic and political weights, design effects range from 1.29 to 2.01, averaging 1.55. These values indicate a moderate increase in variance due to weighting, within the typical range observed for adjusted survey samples. Overall, the application of the weights is therefore recommended when analyzing the dataset, as it effectively adjusts for known deviations from population benchmarks while introducing only a modest reduction in statistical efficiency.

To sum up, while some deviations persist for certain hard-to-reach groups, the raw data demonstrates fairly high accuracy, shows improvement over time, and can be further refined through the application of the provided survey weights. However, data users should exercise some caution when interpreting results for specific subgroups or outcomes directly influenced by factors such as the online mode, undercoverage, or non-response in (political) surveys^21,22^.

### Panel retention and refreshment samples

Panel retention is a critical aspect of longitudinal panel surveys as it can potentially introduce nonresponse bias and thus influence the quality of the data over time^23^. High retention rates also ensure that changes in attitudes and behaviors within respondents can be tracked consistently across waves, while refreshment samples are essential to maintain representativeness, especially when certain demographic groups drop out at higher rates^24^.

**Fig. 3:**
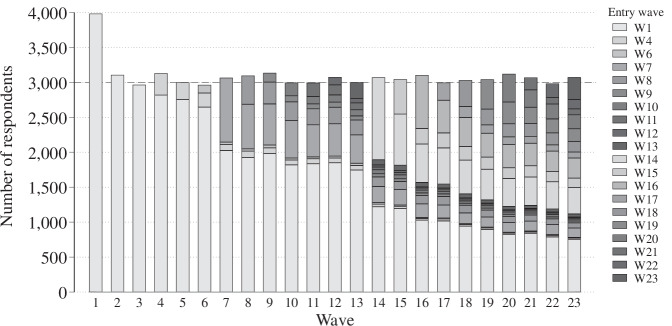
Panel Retention and Refreshment Samples.

Figure 3 illustrates panel retention across the 23 waves of the AUTNES Online Panel Study^4^. Each bar represents the total number of respondents per wave, shaded by the wave in which they entered the panel. The gradual decline in respondents from the initial recruitment (Wave 1, shown in light grey) shows a typical pattern of panel attrition over time. To account for expected attrition while maintaining a sufficiently large sample size, the initial target was set higher at 4,000 respondents, with a 9.0 percent participation rate in the first wave. For subsequent waves, the target was set at 3,000 respondents. Thanks to retention efforts such as repeated invitations and reminders, a significant proportion of panelists remained in the study – even after extended breaks in fieldwork (e.g., between Waves 6 and 7 and Waves 13 and 14) – demonstrating strong participant engagement despite these interruptions.

In addition, continuous targeted recruitment of new respondents took place throughout the panel to address underrepresented demographics and thus ensure that the sample remained representative of the Austrian voting-age population. This combination of retention strategies and refreshment efforts helped to mitigate the impact of attrition and preserve the representativeness of the survey data over the full study period.

Although losses were continuously compensated for with the targeted recruitment of fresh respondents, it is often also of interest to investigate which respondents are more likely to remain in the panel and participate in multiple waves. To address this, we conducted a linear regression analysis to investigate the social and political determinants of panel participation, using the number of waves participated in as the dependent variable and various socio-demographic factors and political predispositions as independent variables. The number of waves ranges from 1 to 23, with a mean of 6.35, meaning that on average respondents took part in about 6 panel waves.

**Fig. 4:**
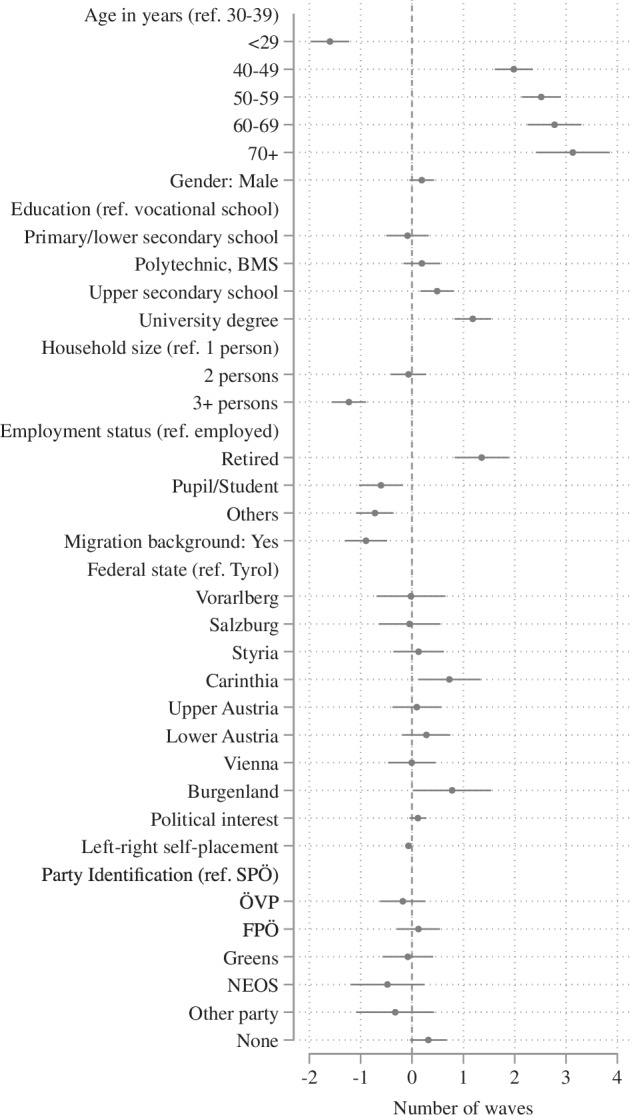
Predictors of Panel Participation.

Figure 4 shows the results of the analysis as a coefficient plot displaying the estimated regression slopes along with 95% confidence intervals. The most pronounced effect is a clear age gradient. Age groups above 40 (40–49, 50–59, 60–69, and 70 + ) participated in approximately two to three more waves than the reference group (30–39), while the youngest age cohort ( < 29) participated in about 1.5 fewer waves. In contrast, gender had no significant effect on wave participation, with the coefficient close to zero. Educational attainment was associated with slightly higher participation, with respondents holding a university degree participating in about one wave more than those with vocational training. Employment status also showed some effects: retired individuals participated in about 1.5 additional waves compared to employed respondents (the reference category), while students and those classified as “others” (e.g., unemployed or inactive) participated in 0.7–0.8 fewer waves. Larger household sizes (3 + persons) and having a migration background were associated with less frequent participation, with about one wave fewer each. Regionally, differences were minimal, with respondents from Carinthia and Burgenland participating slightly more often than those from Tyrol.

Political variables, such as political interest and left-right self-placement, did not significantly influence participation in panel waves. Similarly, no statistically significant effects were found for party identification. Overall, these findings underscore that panel engagement is predominantly influenced by life stage and the associated mobility/stability, rather than by survey-specific or political factors. These patterns closely mirror those reported in other studies on panel attrition, which have also shown that adjustments for attrition in such cases typically have only a minimal impact on the substantive results^25–27^. Considering this, and in light of the continuous recruitment of fresh respondents, we conclude that no major additional adjustments with regard to panel attrition are required in most standard longitudinal analyses.

### Effects of panel participation on response behavior

In addition to panel attrition, a further methodological concern in longitudinal survey research is panel conditioning, i.e., the potential impact of repeated survey participation on respondents’ behavior. While such conditioning may produce positive effects, such as increased familiarity with question formats, it may also lead to reduced diligence, increased satisficing, or heightened attitude extremity over time. As the AUTNES online panel did not include independent cross-sectional samples, we adopted an alternative approach sometimes used in the literature by assessing the association between the number of waves completed and multiple indicators of response quality^28^. This analysis focused on the 2024 post-election wave (Wave 23), at which point some respondents had participated in up to 23 waves, while others had joined the panel more recently, providing substantial variation in survey exposure. To evaluate potential conditioning effects, we regressed several measures of response quality on the number of completed waves. The outcome variables included: (1) an instructed response item (attention check: failed vs. passed); (2) item nonresponse, measured by the number of “don’t know” responses in a matrix battery; (3) straightlining, operationalized as providing identical responses across all items in a grid; (4) attitude extremity, calculated as the average absolute deviation from the scale midpoint; (5) speeding, defined as responding in under two seconds per matrix item^29^; (6) total interview duration (in minutes); and (7) a self-reported measure of diligence, based on agreement with the statement “I completed the questionnaire diligently” on a 5-point Likert scale. The matrix battery from which several of the grid-based indicators were derived focused on immigration attitudes and had been administered in 18 of the 23 waves, making it particularly susceptible to potential panel conditioning effects. To control for confounding influences, all models included the same set of socio-demographic and political variables used in the analysis of panel attrition.

Table 1 shows the results of the analysis. The number of completed panel waves was significantly associated only with a somewhat lower likelihood of failing the attention check, suggesting that more experienced respondents may be more familiar with such instructed response items. For all other indicators—item nonresponse, straightlining, attitude extremity, speeding, interview duration, and self-reported diligence—no significant relationships with panel participation were observed. This indicates that panel conditioning effects are limited in scope and size, with potential positive and negative effects possibly offsetting one another. These findings are consistent with evidence from a recent methodological comparison of the 2024 AUTNES post-election wave with the probability-based Digitize! CSES Post-Election survey, which also found rather minor differences in response quality between the samples^18^. To the extent that conditioning effects may be of concern in specific applications, researchers may consider including the number of completed waves as an additional control variable. Overall, however, the observed effects appear modest.

**Table 1:** Effects of Panel Participation on Response Behavior.

	(1)	(2)	(3)	(4)	(5)	(6)	(7)
	Attention Check: Failed	Item Non-Response	Straight-lining	Attitude Extremity	Speeding	Interview Duration	Self-Reported Diligence
Number of waves	−0.003^**^	0.000	0.001	−0.002	−0.001	3.217	0.004
	(0.001)	(0.002)	(0.001)	(0.001)	(0.001)	(3.910)	(0.002)
*N*	2715	2801	2801	2769	2801	2801	2771
*R*^*2*^	0.084	0.049	0.038	0.143	0.159	0.024	0.140

*Note*: Entries are unstandardized coefficients from linear regression, with standard errors in parentheses. Control variables include age, gender, education, household size, employment status, migration background, federal state, political interest, left-right placement, and party identification – not shown. ^*^p < 0.05, ^**^ p < 0.01, ^***^ p < 0.001.

## Usage Notes

The AUTNES Online Panel Study offers potential for data linkage with other AUTNES datasets available at AUSSDA^4^. Most notably, researchers can combine survey data with data on political party communication and media coverage to conduct comprehensive studies of Austrian political dynamics. The available datasets on party communication include data on party manifestos, press releases, and party Facebook pages. Media coverage datasets include manual content analyses of daily newspapers and automated analyses of TV news, daily newspapers, news websites, and public broadcasting. In addition, longitudinal and cross-sectional datasets from previous AUTNES surveys focusing on the 2008 and 2013 parliamentary elections and the 2014 European Parliament election are available to study trends in public perceptions, political attitudes and behavior. All of these datasets are accessible via the AUTNES Dataverse at https://data.aussda.at/dataverse/autnes.

For the general public, brief analyses and key insights derived from the AUTNES Online Panel Study^4^ are regularly featured on the VIECER blog at https://viecer.univie.ac.at/blog/. These posts provide accessible summaries of findings on voter behavior, political attitudes, and election dynamics, encouraging broader engagement with the data and its implications for Austrian politics. Also, to enhance the accessibility of scientific insights for both political stakeholders and the wider public, an interactive data dashboard^30^ has been made available, enabling users to visualize and explore key variables and trends from the AUTNES Online Panel Study^4^. It can be accessed at: https://autnesdashboard.univie.ac.at:3838/autnes/.

## Data Availability

All code used to produce the analyses for accuracy and panel participation are available on GitHub (https://github.com/juliapartheymueller/autnes/tree/4af7b466b52857edf0438af481f2e27e9023a590/10874_AUTNES_OPS).
